# High hydrostatic pressure extract of garlic increases the HDL cholesterol level via up-regulation of apolipoprotein A-I gene expression in rats fed a high-fat diet

**DOI:** 10.1186/1476-511X-11-77

**Published:** 2012-06-19

**Authors:** Seohyun Lee, Hyunjin Joo, Chong-Tai Kim, In-Hwan Kim, Yangha Kim

**Affiliations:** 1 Department of Nutritional Science and Food Management, Ewha Womans University, Seoul, Republic of Korea; 2 Food Bio-Nano Research Group, Korea Food Research Institute, Seongnam, Gyeonggi, Republic of Korea; 3 Department of Food and Nutrition, College of Health Science, Korea University, Seoul, Republic of Korea

**Keywords:** Garlic, High hydrostatic pressure processing, HDL-cholesterol, Gene expression, Apolipoprotein A-I, ATP-binding cassette transporter A1, Lecithin:cholesterol acyltransferase

## Abstract

**Background:**

Cardiovascular disease (CVD) is the number one cause of mortality worldwide and a low high-density lipoprotein cholesterol (HDL-C) level is an important marker of CVD risk. Garlic (*Allium sativum*) has been widely used in the clinic for treatment of CVD and regulation of lipid metabolism. This study investigated the effects of a high hydrostatic pressure extract of garlic (HEG) on HDL-C level and regulation of hepatic apolipoprotein A-I (apoA-I) gene expression.

**Methods:**

Male Sprague–Dawley rats were divided into two groups and maintained on a high-fat control diet (CON) or high-fat control diet supplemented with high hydrostatic pressure extract of garlic (HEG) for 5 weeks. Changes in the expression of genes related to HDL-C metabolism were analyzed in liver, together with biometric and blood parameters.

**Results:**

In the HEG group, the plasma triglyceride (TG) and low-density lipoprotein cholesterol (LDL-C) levels were significantly decreased in comparison with the CON group (*P* < 0.05). Dietary HEG also lowered the hepatic TG and total cholesterol (TC) levels compared to the CON group. While the plasma HDL-C level and mRNA level of hepatic apoA-I, which is one of primarily proteins of HDL-C particle, were significantly increased in the HEG group compared to the CON group (*P* < 0.05). The gene expression of ATP-binding cassette transporter A1 (ABCA1) and lecithin:cholesterol acyltransferase (LCAT), importantly involved in the biogenesis in HDL, were also up-regulated by dietary HEG.

**Conclusions:**

These results suggest that HEG ameliorates plasma lipid profiles and attenuates hepatic lipid accumulation in the high-fat fed rats. Our findings provides that the effects of HEG on the increase of the plasma HDL-C level was at least partially mediated by up-regulation of hepatic genes expression such as apoA-I, ABCA1, and LCAT in rats fed a high-fat diet.

## Background

Cardiovascular disease (CVD) is one of the major causes of mortality globally, and the number of deaths caused by CVD has been increased at an alarming rate [1]. Dyslipidemia, which includes high levels of blood total cholesterol (TC), low-density lipoprotein cholesterol (LDL-C), and triglyceride (TG), and low level of high-density lipoprotein cholesterol (HDL-C), is considered one of the major risk factors of CVD. Guidelines for the prevention of CVD have focused on decreasing LDL-C levels [2]. However, a substantial proportion of patients with CVD do not have elevated LDL-C levels [3] and the role of other lipoproteins, including HDL, in the progression of CVD and atherosclerosis has been recently investigated [3]. Several epidemiologic studies have reported clinical healthful benefits associated with raising HDL-C level and prevention of CVD [4-7]. Therefore, regulation of HDL-C level should be one of the main goals in the treatment of atherogenic dyslipidemia and reduction of the risk of CVD. Apolipoprotein A-I (apoA-I) is a major structural protein constituent in HDL particles, and plays an important role in regulating HDL biosynthesis [8]. Also, ATP-binding cassette transporter A1 (ABCA1) and lecithin:cholesterol acyltransferase (LCAT) directly contributes to HDL biogenesis [9].

Garlic (*Allium sativum*) is one of the most well-known herbal medicines worldwide and there has been increasing interest in using garlic as a cholesterol-lowering agent. However, the effect of garlic consumption on HDL-C level remains controversial and many studies have reported conflicting results regarding this relationship [10-12]. The major components of garlic are water, carbohydrates, proteins, fat, dietary fiber, and essential amino acids. It also contains high levels of saponins, vitamins, minerals, and various biologically active sulfur compounds such as alliin, allicin, ajoene, diallyl disulfide, allyl methanethiosulfinate, diallyltrisulfide, and S- allylcysteine [13,14]. However, these bioactive compounds of garlic are affected by processing methods; thus, the processing technique may be the main reason for the differences in the reported efficacy of various garlic preparations.

High hydrostatic pressure processing (HPP) is a method used to reduce microbial counts, and has been shown to affect enzyme activity and food product functionality since covalent bonds are not affected by pressure [15]. Thus, HPP may only minimally modify the nutritional value and sensory quality of fruits and vegetables. A recent study reported that HPP affected the content, category and intensity of the volatile compounds in garlic by decreasing the alliinase activity, which influenced the formation of allicin [16]. Furthermore, the effects of HPP on the total polyphenols, total flavonoid, antioxidant activities, and 5-hydroxymethyl-2-furaldehyde (5-HMF) contents in garlic were previously examined [17]. The objective of this study was to elucidate the antidyslipidemic effect of the high hydrostatic pressure extract of garlic (HEG), produced by HPP, in rats fed a high-fat diet. This effect could be explained by alteration of the expression of genes that is involved in cholesterol metabolism. We hypothesized that HEG may increase concentration of blood HDL-C level via induction of genes expression, related with HDL biogenesis, such as apoA-I, ABCA1, and LCAT in rats fed a high-fat diet.

## Results

### Body weight and energy intake

**Figure 1:**
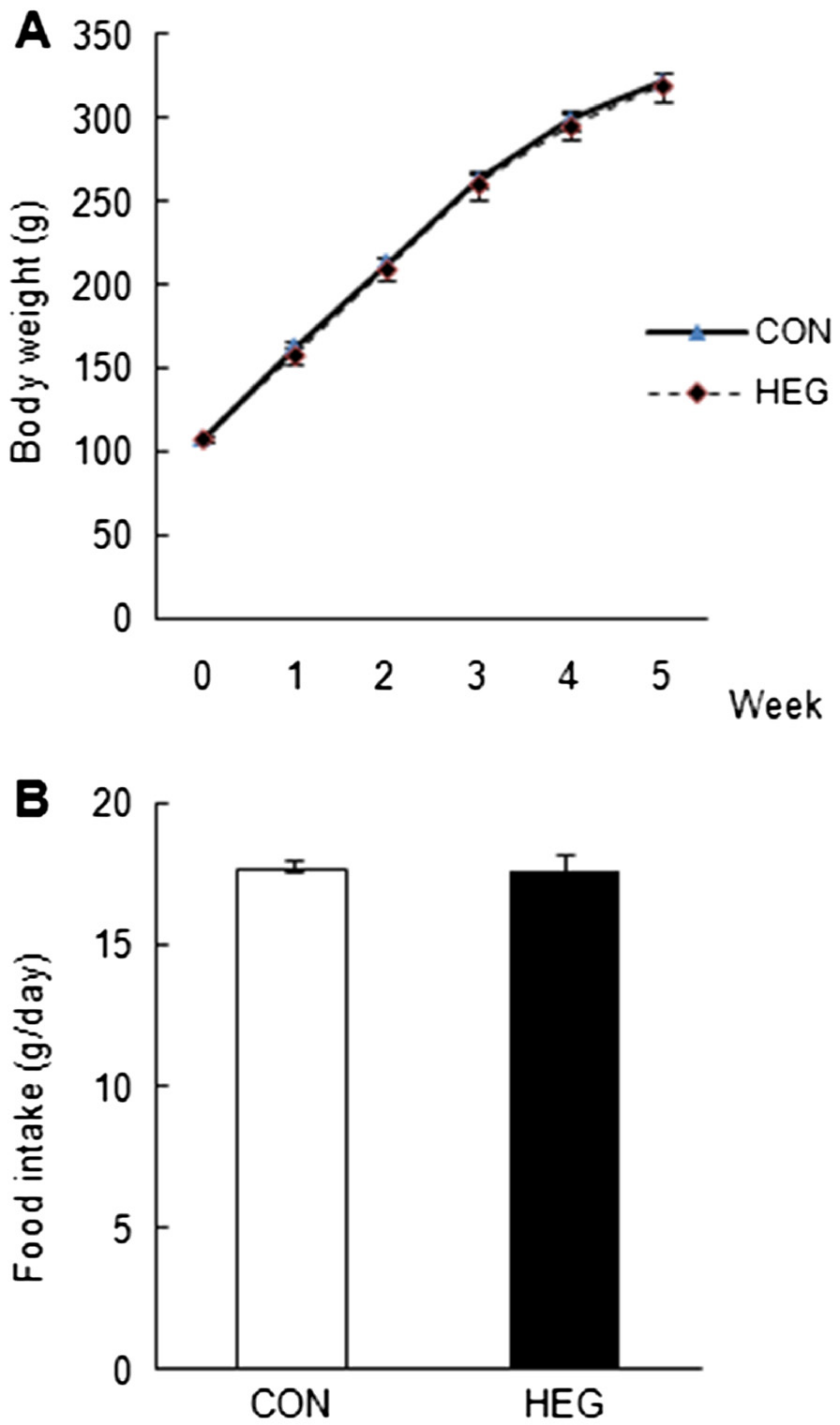
**Effects of HEG supplementation on (A) body weight and (B) food intake.** Body weight and food intake were measured twice a week. Values are expressed as mean ± SEM (n = 10).

Rats were assigned randomly to two experimental groups and the initial body weight of the rats was not significantly different between the two groups (Table 1). Final body weight was also not significantly different between the CON and HEG groups (Figure 1A; Table 1). There were no significant differences in the amount of energy intake and energy efficiency ratio between groups over the experimental period (Table 1).

**Table 1:** Effect of dietary HEG supplementation on physiological variables

**Variables**	**CON**	**HEG**
Initial body weight (g)	107.43 ± 1.52	108.04 ± 1.51
Final body weight (g)	321.40 ± 6.25	319.25 ± 8.63
Food intake (g/day)	17.58 ± 0.41	17.56 ± 0.61
Energy intake (kcal/day)	81.64 ± 1.91	80.29 ± 2.78
Energy efficiency ratio (EER)^1)^	0.74 ± 0.00	0.74 ± 0.00
Liver weight	3.01 ± 0.06	3.08 ± 0.07
(g/100 g body weight)		
Epididymal adipose tissue weight	1.24 ± 0.05	1.28 ± 0.07
(g/100 g body weight)		

Values are expressed as mean ± SEM; n = 10 for each treatment group.

^1)^ Energy efficiency ratio (EER) = Body weight gain (g/day)/Energy intake (kcal/day).

### Liver weight and plasma AST and ALT activities

At the doses given, HEG did not cause a rise in plasma AST and ALT activities when compared with those of CON group (Table 2). In addition, the liver weight (Table 1) was unaffected by HEG treatment. These data indicate that the HEG was well tolerated by the rats (Table 2).

**Table 2:** Effects of dietary HEG supplementation on plasma and hepatic lipid profiles

	**CON**	**HEG**
**Plasma**		
Total cholesterol (mg/dl)	70.26 ± 2.31	68.57 ± 4.60
LDL cholesterol (mg/dl)	34.97 ± 2.76	26.49 ± 3.40^*^
HDL cholesterol (mg/dl)	25.42 ± 1.21	40.35 ± 6.30^*^
Triglyceride (mg/dl)	50.37 ± 3.51	40.64 ± 3.94^*^
LDL-C/HDL-C	2.60 ± 1.20	1.97 ± 0.26^*^
HDL-C/TC	0.37 ± 0.02	0.53 ± 0.05^*^
Atherogenic Index	1.75 ± 0.12	0.97 ± 0.18^*^
AST (IU/L)	65.45 ± 8.29	45.07 ± 3.98^*^
ALT (IU/L)	9.07 ± 0.79	10.69 ± 1.37
**Hepatic lipids**		
Triglyceride (mg/g liver)	6.46 ± 0.65	4.18 ± 0.68^*^
Total cholesterol (mg/g liver)	1.70 ± 0.09	0.97 ± 0.11^*^

Values are expressed as mean ± SEM; n = 10 for each treatment group.

^*^: Significantly different from the CON group at *P* < 0.05 by Student’s *t*-test.

### Plasma lipid profiles

The HEG group had significantly lower plasma concentrations of TG and LDL-C by 19% and 24%, respectively, in comparison with the CON group (Table 2). The atherogenic index (AI) and the ratio of LDL-C to HDL-C, which are all significant risk factors of CVD, were also significantly decreased by 45% and 24%, respectively, in the HEG group as compared to the CON group (Table 2). While the HDL-C level and the ratio of HDL-C to TC were significantly increased by 59% and 43%, respectively, in the HEG group relative to the CON group in rats fed a high-fat diet (Table 2).

### Hepatic lipid profiles

The contents of hepatic TG and TC were investigated to evaluate the effects of HEG on the reduction of hepatic lipids levels. The amounts of hepatic TC and TG were significantly reduced by 43% and 35%, respectively, in the HEG group as compared to the CON group (Table 2). These results support the hypothesis that HEG may regulate TG and TC levels in rats fed a high-fat diet.

### Gene expressions of hepatic apoA-1, ABCA1, and LCAT

**Figure 2:**
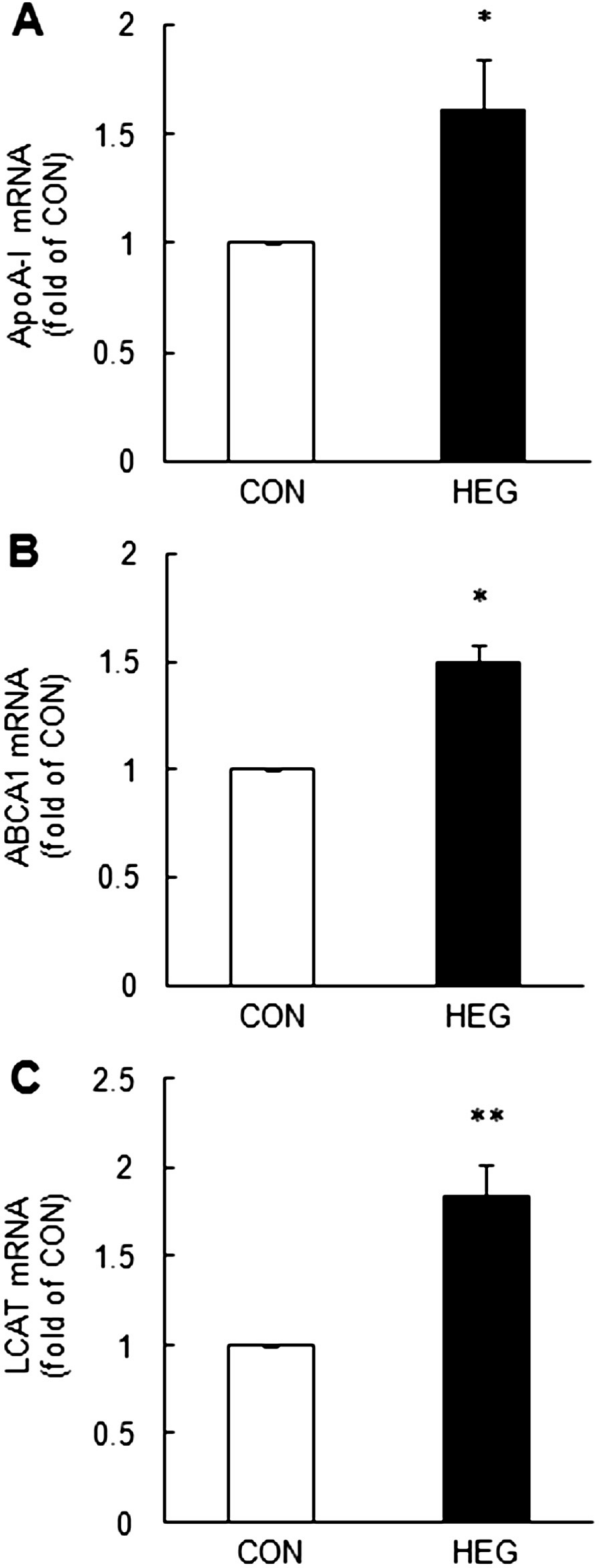
**Effects of HEG supplementation on the hepatic apoA-I (A), ABCA1 (B), and LCAT (C) mRNA levels.** Total RNA extracted from the liver was used for mRNA expression analysis by real-time PCR. Bars are expressed as mean ± SEM (n = 10). * *P* < 0.05 and ** *P* < 0.01 *versus* the CON group.

ApoA-I is one of major structural protein constituents in HDL particle and alteration of the hepatic apoA-I gene expression affects blood apoA-I concentration. Thus, we investigated the effect of HEG supplementation on the mRNA level of apoA-I. Our data showed that the hepatic apoA-I mRNA level was 1.6- fold higher in the HEG group than in the CON group (*P* < 0.05) (Figure 2A). The mRNA levels of hepatic ABCA1 and LCAT, which are importantly involved in the HDL biogenesis, were also significantly increased by 1.5- fold (*P* < 0.05) and 1.8- fold (*P* < 0.01), respectively, in the HEG group as compared as the CON group (Figure 2B, C) in high-fat fed rats.

## Discussion

We investigated the effects of HEG on the regulation of lipid profiles in rats fed a high-fat diet. The antilipidemic effects of garlic have been previously reported [18-20]. Our results showed that dietary HEG reduced hepatic TC and TG levels. Previous studies have reported that garlic reduces hepatic cholesterol and TG levels [21-23]. The hypocholesterolemic effects of the garlic extract stem occurred in part from the inhibition of hepatic cholesterol synthesis by water-soluble sulfur compounds, such as S-allylcysteine and ajoene [22]. Reduction of hepatic cholesterol may also be due to inhibition of cholesterol biosynthesis or activation of reverse cholesterol transport (RCT), which extracts cholesterol. Also, *in vivo* and *in vitro* studies have indicated that garlic and its constituents inhibit pivotal enzymes involved in the biosynthesis of cholesterol, such as HMG-CoA reductase [9,18,22].

In the present study, treatment with HEG reduced plasma TG concentration in rats fed a high-fat diet. This observation is in agreement with human [23,24] and animal [18] studies, which showed an inverse association between dietary garlic and the concentration of plasma TG. Also, studies using cell lines and animal models have shown that garlic has beneficial effects on reducing the accumulation of TG in blood. These effects were suggested with activation of liver phosphatidate phosphohydrolase (PAP) and fatty acid synthease (FAS), which play a critical role in controlling the biosynthesis of TG [21,25,26].

Dietary HEG increased plasma HDL-C concentration and decreased plasma LDL-C concentration. The primary function of HDL is to remove cholesterol from peripheral cells and transport cholesterol to the liver for biliary excretion by RCT. In addition, HDL has been shown to prevent foam cell formation, thus retarding the progress of atherosclerosis [27]. Therefore, the present results clearly demonstrated that supplementation of HEG favorably modified cholesterol metabolism. Our findings were in agreement with other studies, which reported that garlic consumption increased HDL-C level in hyperlipidemic individuals [10], animals [11], and patients with coronary artery disease [28]. The LDL-C/HDL-C ratio and HDL-C/TC ratio are important indicators of CVD and atherosclerosis [29]. In this study, the HDL-C/TC ratio was higher and the LDL-C/HDL-C ratio was lower in the HEG group relative to the CON group. When garlic powder was added into the diet at a concentration of 1%, plasma TC and LDL-C levels were decreased and HDL-C level were increased in rabbits [30], resulting in a decrease in the LDL-C/HDL-C ratio. Hence, our observations indicate that dietary HEG may have hypolipidemic effects by reducing the TC and TG levels in the body and CVD protection effects by regulating the HDL-C/TC and LDL-C/HDL-C ratios.

ApoA-I is a main protein component of HDL particles and constitutes about 70% of the apolipoprotein content of HDL particles [31], and is thought to be a better predictor of CVD risk than TC and LDL-C [32]. ApoA-I acts as a cofactor for LCAT, which is a key enzyme involved in removing excess cholesterol from tissues and incorporating it into the HDL [33]. Hence, plasma apoA-I concentration is correlated with plasma HDL-C concentration, and apoA-I mRNA level may be an important determinant of blood HDL-C concentration [34,35]. However, less is known about the effect of garlic on the HDL-C and the expression of genes associated with HDL-C metabolism.

Treatment with HEG increased the apoA-I mRNA level, which corresponded to an increase in the HDL-C level. Dorfman *et al*. [36] reported that treatment with dietary fatty acids, such as coconut oil and butter, resulted in an increase in both the blood HDL-C level and apoA-I concentrations, and these effects were found to be associated with the expression of apoA-I gene in animal studies. They indicate that regulation of apoA-I gene expression may be associated with the regulation of HDL-C level. Therefore, we suggest cautiously that dietary HEG supplementation may increase the HDL-C level with up-regulation of the apoA-I gene expression. Previous studies have shown that garlic consumption produces hypolipidemic effects without changing the blood HDL-C level [37]. Furthermore, although the apoA-I mRNA level is associated with HDL-C level, the increased level of HDL-C is not always caused by the change of hepatic apoA-I mRNA level. Choi *et al.*[38] reported that soy pinitol supplementation increased plasma HDL-C and apoA-I concentrations, whereas the expression of the hepatic apoA-I gene was not affected by soy pinitol. However, in our study, HEG supplementation was shown to increase the plasma HDL-C concentration with up-regulation of apoA-I gene expression.

ApoA-I plays a pivotal role in RCT by functioning as an acceptor of cellular phospholipids and cholesterol and as an activator of LCAT. Recent studies indicated that the functional interactions of apoA-I with ABCA1 is necessary for the initial lipidation of apoA-I [9]. ABCA1 facilitates the efflux of cellular phospholipids and cholesterol to extracellular lipid-poor apoA-I, and lapidated apoA-I produces discoidal preβ-HDL particles. Thus, interaction between apoA-I and ABCA1 is an essential step for formation of HDL that ultimately determines plasma HDL-C level. The preβ-HDL particles are subsequently transformed into mature spherical HDL particles by LCAT, which is an enzyme that concerts free cholesterol into cholesteryl ester and is critical for the maintenance and maturation of HDL metabolism [9]. In this study, we have showed that the expressions of hepatic ABCA1 and LCAT were increased by HEG supplementation.

## Conclusions

In conclusion, HEG ameliorated plasma lipid profiles by decreasing the LDL-C level and increasing the HDL-C level in high-fat fed rats. Also dietary supplementation with HEG attenuated hepatic lipid accumulation by reducing the contents of TG and TC. This study provides that the effects of HEG on the increase of the plasma HDL-C level was at least partially mediated by up-regulation of genes related with HDL biogenesis, such as apoA-I, ABCA1, and LCAT. Accordingly, the antidyslipidemic effects of HEG imply that HEG has potential as a bioactive food ingredient for the prevention of CVD in rats fed a high-fat diet.

## Methods

### Materials

High hydrostatic pressure extract of garlic (HEG) was provided as a gift from the Korea Food Research Institute (Seongnam, Gyeonggi, Korea). The dietary HEG powder used in this study was prepared as follows: Cloves of fresh garlic were purchased from the local market (Seosan, Chungnam, Korea). 550 g of the fresh garlic was crushed with 450 ml of distilled water and digested with the following enzymes: Viscozyme L, Celluclast 1.5 L, and Liquozyme supra (Novo Nordisk, Bagsvaerd, Denmark). The garlic slurry was adjusted to pH 5.5 and processed under 100 MPa for a 24 hr at 50°C using a high hydrostatic pressure machine TFS-2 L (Toyo Koatsu Co. Ltd., Hiroshima, Japan) to solubilize the garlic cell wall and to obtain the major component such as allicin and water soluble materials. The HEG contained 1.24 mg/g of allicin, which was higher than hot water extract of garlic (0.86 mg/g) as determined by high performance liquid chromatography. The hot water extract of garlic was prepared from the grounded garlic boiled in 100°C hot water for 1 hr. All other chemicals were of the highest pure grade available.

### Animals and diets

Four–week–old male Sprague–Dawley (SD) rats (G-bio company, Gyeonggi, Korea), weighing 60 ~ 70 g, were individually housed in temperature-controlled room (22 ± 2°C) under 12 hr light/dark conditions. Rats consumed a commercial chow diet (Zeigler Bros Inc., PA, USA) and water *ad libitum* for a week to stabilize their metabolic condition. After acclimatization, the rats were completely randomized into two groups of ten rats each and maintained on one of the following diets: a high-fat control diet (CON) or a high-fat control diet supplemented with 2% (w/w) high hydrostatic pressure extract of garlic (HEG) for 5 weeks. To induce hyperlipidemia in the rats, they were maintained on a high-fat control diet (45% of total energy as fat) based on the AIN-93 G diet with slight modifications [39] (Table 3). The amount of corn starch in the CON was adjusted accordingly when the dietary HEG powder was added (Table 3). Food intake and body weight were measured twice a week during the treatment period (Monday, Thursday). The average weekly body weight and daily food intake of two groups were calculated. At the end of the experiment, the rats were deprived of food overnight and anesthetized with intramuscular injection of Zoletil/Rompun (Bayer, Leverkusen, Germany) mixture (4:1) at a dose of 0.1 ml/ 80 g body weight. A central longitudinal incision was made in the abdominal wall and blood samples were collected by cardiac puncture. Blood samples were centrifuged at 2800 × *g* for 20 min at 4°C to obtain plasma, which was then stored at −20°C for biochemical analyses. The liver was harvested, frozen immediately in liquid nitrogen, and stored in −70°C. All experimental protocols were approved by The Ewha Womans University Animal Experimentation Ethics Committee for the care and use of laboratory animals.

**Table 3:** Composition of experimental diets (g/kg diet)

**Ingredient**	**CON**	**HEG**
Casein	170.73	170.73
Sucrose	121.95	121.95
Corn Starch	201.71	181.71
Dyetrose	155.00	155.00
L-Cystine	2.20	2.20
Cellulose	60.98	60.98
Lard	229.50	229.50
Mineral Mix^1)^	42.68	42.68
Vitamin Mix^2)^	12.20	12.20
Choline Bitartrate	3.05	3.05
High hydrostatic pressure extract of garlic^3)^	-	20.00
**Fat% (calories)**	45	45
**Total**	1000	1000

^1)^ AIN-93 G mineral mixture.

^2)^ AIN-93 G vitamin mixture.

^3)^ The amount of corn starch in control diet (CON) was adjusted accordingly when the dietary high hydrostatic pressure extract of garlic (HEG) was added.

### Plasma biochemical measurements

The plasma levels of TG, TC, HDL-C, AST and ALT were determined by the enzymatic colorimetric methods [40] using commercial kits (Asan Pharm Co., Ltd., Seoul, Korea). The Atherogenic Index (AI) was calculated using the formula of Rosenfeld [41]. In addition, the ratio of HDL-C to TC was calculated.

$$*\text{AI}=\left(\text{TC}-\text{HDL}-\text{cholesterol}\right)/\text{HDL}-\text{cholesterol}$$

### Hepatic and fecal lipids analysis

Hepatic and fecal lipids were extracted using the method described by Bligh and Dyer [42]. TC and TG were determined by enzymatic colorimetric methods using commercial kits as described above.

### Real-time qRT-PCR

Total RNA was isolated from liver tissue using the Trizol reagent (Invitrogen, Carlsbud, CA, USA). The corresponding cDNA was synthesized from 4 μg of total RNA using M-MLV reverse transcriptase. After cDNA synthesis, real-time qPCR was performed using universal SYBR Green PCR Master Mix on a fluorometric thermal cycler (Rotor-Gene 2000, Corbett Research, Mortlake, Australia). Primers were designed using an online program (Primer3; http://frodo.wi.mit.edu/) [43]. The sequences of the sense and antisense primers used for amplification were as follows: apoA-I, 5′-CTGGGTTCAACTGTTGGTCG-3′ and 5′-GGGCTGCATCTTCTGTTTCA-3′; ABCA1, 5′- CCCAATCCCAAACACTCCTT-3′ and 5′-TCTTCATCGTCCAGTTCCCA-3′; LCAT, 5′- TAACAATGGGTATGTGCGGG-3′ and 5′-GCCAAGGCTGTGTCCAATAA-3′; β-actin, 5′-GGCACCACACTTTCTACAAT-3′ and 5′-AGGTCTCAAACATGATCTGG-3′. The delta delta C_t_ method was used for relative quantification [44]. The delta C_t_ value for each sample was determined by calculating the difference between the C_t_ value of the target gene and the C_t_ value of the β-actin reference gene. The normalized level of expression of the target gene in each sample was calculated using the formula 2^―∆∆Ct^. Values were expressed as fold change over the control.

### Statistical analysis

The effects of HEG on the plasma lipid concentrations and hepatic lipid contents were analyzed. All data are presented as mean ± standard error of mean (SEM, n = number of rats). The statistical significances of the difference between the means of the two groups of samples (CON and HEG) were analyzed using Student’s *t*-test. Statistical analyses were performed using the SAS software version 9.2 (SAS Inc., NC, USA). *P* < 0.05 and *P* < 0.01 were taken to indicate a significant difference.

## Abbreviations

ABCA1: ATP-binding cassette transporter A1; AI: Atherogenic index; ApoA-I: Apolipoprotein A-I; CON: High-fat control diet; CVD: Cardiovascular diseases; FAS: Fatty acid synthease; HDL-C: High-density lipoprotein cholesterol; HEG: High Hydrostatic pressure extract of garlic; HPP: High hydrostatic pressure processing; LCAT: Lecithin:cholesterol acyltransferase; LDL-C: Low-density lipoprotein cholesterol; PAP: Phosphatidate phosphohydrolase; RCT: Reverse cholesterol transport; TC: Total cholesterol; TG: Triglyceride.

## Competing interests

The authors declare that they have no competing interests.

## Authors’ contributions

SL and YK conceived the study and its design. SL wrote the manuscript, carried out all experiments, and analyzed data. HJ participated in the experiment for gene expression analysis. CK and IK contributed critical review, intellectual input in discussion and overall presentation of paper. YK refined the final draft and revised of this manuscript for publication. All authors read and approved the final manuscript.
